# Arming Technology in Yeast—Novel Strategy for Whole-cell Biocatalyst and Protein Engineering

**DOI:** 10.3390/biom3030632

**Published:** 2013-09-09

**Authors:** Kouichi Kuroda, Mitsuyoshi Ueda

**Affiliations:** Division of Applied Life Sciences, Graduate School of Agriculture, Kyoto University, Sakyo-ku, Kyoto 606-8502, Japan; E-Mail: k_kuro@kais.kyoto-u.ac.jp

**Keywords:** cell surface engineering, arming yeast, whole-cell biocatalyst, molecular display, arming technology, cell surface display, protein engineering, directed evolution, combinatorial bioengineering, single cell analysis

## Abstract

Cell surface display of proteins/peptides, in contrast to the conventional intracellular expression, has many attractive features. This arming technology is especially effective when yeasts are used as a host, because eukaryotic modifications that are often required for functional use can be added to the surface-displayed proteins/peptides. A part of various cell wall or plasma membrane proteins can be genetically fused to the proteins/peptides of interest to be displayed. This technology, leading to the generation of so-called “arming technology”, can be employed for basic and applied research purposes. In this article, we describe various strategies for the construction of arming yeasts, and outline the diverse applications of this technology to industrial processes such as biofuel and chemical productions, pollutant removal, and health-related processes, including oral vaccines. In addition, arming technology is suitable for protein engineering and directed evolution through high-throughput screening that is made possible by the feature that proteins/peptides displayed on cell surface can be directly analyzed using intact cells without concentration and purification. Actually, novel proteins/peptides with improved or developed functions have been created, and development of diagnostic/therapeutic antibodies are likely to benefit from this powerful approach.

## 1. Introduction

Arming technology in yeast using the cell surface display system is an innovative technology for the construction of whole-cell biocatalysts and protein engineering through high-throughput screening of protein libraries. Although intracellular expression and extracellular secretion have been employed in conventional strategies for molecular breeding, the cell surface (including the cell wall and plasma membrane) is an attractive location for heterologous gene expression. Proteins localized at the cell surface play an important role in signal transduction, recognition and transport of environmental substances, morphology formation, and various other reactions. In arming technology, functional heterologous proteins/peptides are genetically immobilized on the cell surface by fusion with the domain for cell wall- or plasma membrane-anchoring domains. Cell surface-engineered yeasts constructed using the arming technology, have been termed “arming yeasts” [1,2,3]. Arming technology has advantages that are not found in conventional intracellular expression and extracellular secretion approaches. Immobilization on the cell surface itself retains the features of immobilized enzymes, and increases the thermal stability of the displayed proteins/peptides [4,5]. Preparation of protein/peptide-displaying cells can be achieved only by cell cultivation, in which the two processes of production of proteins/peptides and immobilization on cell surface are simultaneously undertaken, leading to cost effectiveness. Furthermore, the displayed proteins/peptides can interact with environmental substances with high molecular mass that cannot be imported into a cell. Surface-engineered cells prepared by cell cultivation are ready to use as microparticles covered by proteins/peptides, whereas troublesome protein purification and concentration procedures are required for the analysis and subsequent application of proteins produced by intracellular expression and extracellular secretion. Therefore, arming technology is also suitable for high-throughput screening of proteins/peptides from the mutant library at the single-cell level, and amino acid sequences of the screened proteins/peptides can be easily determined by DNA sequencing of the introduced plasmid [6,7,8,9,10]. Therefore, arming technology has been applied to wide range of targets such as whole-cell biocatalysts, bioremediation, biosensors, live vaccines, high-throughput screening of ligand peptides for receptors, and protein engineering [2,3,11,12,13,14].

The cell surface display system has also been established in gram-negative bacteria, gram-positive bacteria, and phages [11,15,16,17,18]. So far, many proteins/peptides have been successfully displayed with maintenance of their functions. In contrast to bacteria and phages, yeasts are equipped with the quality control and modification systems of eukaryotic secretory pathways. Therefore, in the case of target proteins that have a high molecular mass or require glycosylation modification, yeasts are suitable hosts for cell surface display. In addition, simultaneous display of multiple kinds of proteins/peptides on the same cell surface can be performed in yeasts by using different auxotrophic markers, leading to the enhanced potential of surface-engineered yeasts. Recently, the cell surface display system has also been developed in *Aspergillus oryzae* as a eukaryote except yeasts [19,20]. Here, we review the advances of molecular breeding of novel cells and protein engineering by arming technology in yeasts such as *Saccharomyces cerevisiae*, *Pichia pastoris*, and *Yarrowia lipolytica*.

## 2. Cell Surface Display System in Yeast

**Figure 1:**
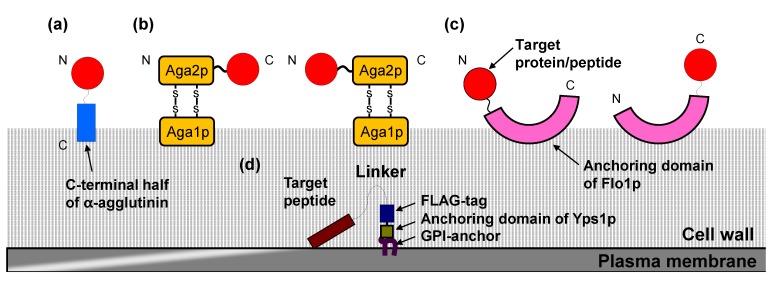
Cell surface display system in *S. cerevisiae* (**a**) α-agglutinin-based display system; (**b**) **a**-agglutinin-based display system; (**c**) Flo1p-based display system; (**d**) Membrane display system by anchoring domain of Yps1p.

Among yeasts, arming technology was first developed in *S. cerevisiae*. Subsequently, based on the success of this approach using full length or cell wall-anchoring domains of cell wall proteins from *S. cerevisiae*, similar display systems have been transferred into other yeasts, including *P. pastoris* and *Y. lipolytica*. The cell surface display systems (Figure 1) are classified into two systems. One is the *N*-terminus free display in which target proteins/peptides are produced as fusions with the secretion signal sequence at the *N*-terminus and the cell wall-anchoring domain at the *C*-terminus. The other is the *C*-terminus free display, in which secretion signal sequence, cell wall-anchoring domain, and target proteins/peptides are fused in this order. The effect of the orientation on the display efficiency and on the functional properties depends on the kinds of target proteins/peptides to be displayed. Therefore, the fusion order itself, namely whether the *N*- or *C*-terminus of target proteins/peptides is fused with cell wall-anchoring domain, is important. In addition, the length of spacer peptides between target proteins/peptides and the cell wall-anchoring domain is also important. By optimizing the spacer length, there are cases in which the function of displayed proteins/peptides has been improved [21,22].

### 2.1. Cell Surface Display System in S. cerevisiae

*S. cerevisiae* has the “generally regarded as safe” (GRAS) approval of the Food and Drug Administration, and thus is suitable for industrial use. Among the cell wall proteins that are linked to the glucan layer by a covalent bond in yeast, α-agglutinin and **a**-agglutinin are representatives of cell wall-anchoring proteins used for cell surface display of target proteins/peptides. These proteins are involved in sexual adhesion of mating type α and **a** cells, and have characteristics specific to cell wall proteins. Specifically, they possess an *N*-terminal secretion signal sequence for transportation to the cell surface and *C*-terminal glycosylphosphatidylinositol (GPI) anchor attachment signal sequence for transient anchoring in the plasma membrane [23]. GPI-anchored proteins in the plasma membrane are released via cleavage by phosphatidylinositol-specific phospholipase C (PI-PLC), and then bind to the cell wall with a covalent bond by cell wall-anchoring domain. For the *N*-terminus free display system, α-agglutinin is the most frequently used approach (Figure 1a). Many proteins/peptides, including those with relatively large molecular masses and glycosylation requirements, have been successfully displayed on the yeast cell surface using the cell wall-anchoring domain of α-agglutinin [3,13,14,24]. In the α-agglutinin-based display system, target proteins/peptides are fused to the secretion signal sequence at the *N*-terminus and to the cell wall-anchoring domain (including the GPI anchor attachment signal sequence) at the *C*-terminus. In addition, Flo1p, which is a GPI-anchored cell wall protein involved in flocculation, is available for the *N*-terminal free display in a similar manner (Figure 1c) [25]. Other GPI-anchored cell wall proteins (Cwp1p, Cwp2p, Tip1p, Sed1p, YCR89w, and Tir1p) have the potential as cell wall-anchoring domains, although they are less frequently used because of the past successful performance of α- and **a**-agglutinin-based display system [26].

On the other hand, **a**-agglutinin has been used in the *C*-terminus free display system (Figure 1b). **a**-Agglutinin consists of Aga1p and Aga2p subunits [7]. The secreted Aga2p subunit is linked to the Aga1p subunit via two disulfide bonds, which are incorporated in the cell wall. Therefore, in the **a**-agglutinin-based display system, target proteins/peptides are fused with the *C*-terminus of Aga2p. In addition, this system has also been used in the *N*-terminal free display by fusing target proteins/peptides with the *N*-terminus of Aga2p [27]. Flo1p is also available for the *C*-terminus free display, in which truncated Flo1p without GPI anchor attachment signal sequence is used as an adhesive region (Figure 1c) [28]. Pir proteins (Pir1–4p) have been used as anchoring proteins in the *C*-terminus free display, and this permits extraction of displayed proteins/peptides from the cell wall by alkali treatment [29].

Several strategies have been employed to improve the efficiency of cell surface display in *S. cerevisiae*. Vector engineering by high copy number plasmid and the improvement of host strain enhanced the efficiency in the α-agglutinin-based display system [30]. Additionally, screening from a cDNA library identified five genes whose overexpression improved the efficiency of the **a**-agglutinin-based display system [31].

### 2.2. Cell Surface Display System in P. pastoris

The methylotrophic strain *P. pastoris* can grow on an economical carbon source and allows high-density culture. Therefore, it is also a suitable host for use in large-scale fermentation cultures of surface-engineered cells. In *P. pastoris*, both the *N*- and *C*-terminus-free display systems have been established using cell wall proteins (α-agglutinin, **a**-agglutinin, Flo1p, Pir1p, Sed1p, and Tip1p) from *S. cerevisiae* in the same strategy as outlined above [32,33,34].

### 2.3. Cell Surface Display System in Y. lipolytica

The oleaginous yeast *Y. lipolytica* is a heterothallic and dimorphic yeast, and has the high potential for secreting heterologous proteins, which is a preferred feature in industrial uses. Several cell wall proteins in *Y. lipolytica* have been identified. YlCWP1 and YlPIR1 from *Y. lipolytica*, which are GPI-anchored cell wall proteins, allow *N*-terminus free display and *C*-terminus free display, respectively [35].

### 2.4. Membrane Display System in S. cerevisiae

Almost all displayed proteins/peptides in yeasts are localized to the cell wall as described above. However, the display of proteins/peptides on the plasma membrane is desirable when there is a requirement for interaction with membrane proteins such as receptors. Membrane display of proteins/peptides is performed using the anchoring domain of Yps1p, a GPI-anchored plasma membrane protein. The fusion of this domain with the *C*-terminus of the peptide ligand allows the display on the plasma membrane and activation of either endogenous or heterologous G protein-coupled receptors (GPCRs) in *S. cerevisiae* [36,37].

## 3. Whole-Cell Biocatalyst by Arming Technology

### 3.1. Biofuel Production

**Figure 2:**
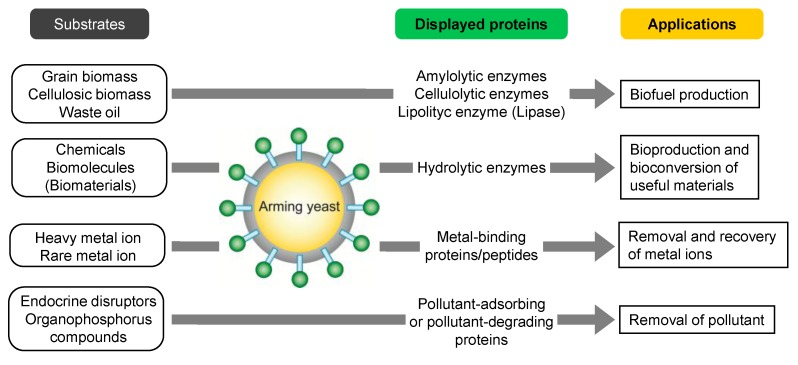
Whole-cell biocatalyst constructed by arming technology and their applications.

Development of whole-cell biocatalysts to produce biofuel from biomass such as grain or cellulosic biomass has attracted attention as a means of creating a sustainable society based on biomass resources. Especially, consolidated bioprocessing (CBP) of lignocellulose to ethanol is an ideal system combining all processes such as enzyme production, hydrolytic degradation, and fermentation of sugar [38]. The arming technology has been applied to yeasts for the construction of whole-cell biocatalysts that can perform saccharification and fermentation. Starch and cellulose are major components in grain and cellulosic biomass, respectively. Therefore, the cell surface display of enzymes for hydrolytic degradation of these components was attempted in order to achieve CBP (Figure 2). As a starch-degrading enzyme, an exotype glucoamylase from *Rhizopus oryzae* was displayed on *S. cerevisiae* using the α-agglutinin-based display system, which allowed the direct production of ethanol from starch through the saccharification of starch on the cell surface and the subsequent fermentation of released glucose [39]. In addition to glucoamylase, an endotype α-amylase from *Bacillus stearothermophilus* was co-displayed with glucoamylase, leading to an improved production efficiency of ethanol from starch [40]. In the same way, α-amylase from *Streptococcus bovis* was also displayed using the Flo1p-based display system on glucoamylase-displaying yeast constructed by the α-agglutinin-based display system [41]. The arming yeasts described above show improved ethanol production and faster growth in the medium including starch as the sole carbon source.

For degradation of cellulose, various enzymes including endoglucanases (EGs), cellobiohydrolases (CBHs), and β-glucosidases (BGLs) are necessary. Therefore, the potential of co-display on the same cell surface is an important feature in yeast, which is suitable for the reaction by multiple enzymes. Actually, the co-display of carboxymethylcellulase (CMCase) and BGL1 from *Aspergillus aculeatus* on *S. cerevisiae* enabled assimilation of cellobiose or oligosaccharide and growth in medium containing these materials as the sole carbon source [42]. EG acts randomly against the amorphous region of the cellulose chain to produce reducing and nonreducing ends, and CBH releases cellobiose from both ends. Therefore, efficient degradation of cellulose to cellobiose and cellooligosaccharides is achieved by the endo-exo synergism of EG and CBH. Finally, BGL hydrolyzes the generated cello-oligosaccharides to glucose. The arming yeast constructed by co-display of endoglucanase II (EG II) and cellobiohydrolase II (CBH II) from *Trichoderma reesei* and BGL1 from *A. aculeatus* can produce ethanol directly from phosphoric-acid-swollen cellulose due to the combined activities of these three cell surface enzymes [43]. In sake yeast, ethanol production from β-glucan can be achieved by cell surface display of EG and BGL from *A. oryzae* [44]. Furthermore, in arming yeast co-displaying EG II, CBH II, and BGL1, four non-conserved amino acids in the carbohydrate-binding module (CBM) of EG II were comprehensively mutated. In the case of co-displaying multiple kinds of enzymes on the same cell surface, the control or design of the display ratio of enzymes is a next challenge in arming technology. Recently, some approaches to this challenge were performed for optimized synergistic effects of displayed enzymes [45,46]. The optimal combination of yeasts displaying EG II with mutated CBMs was determined by inoculating a yeast library into selection liquid medium that included newspaper as the sole carbon source. The selected yeast mixture showed the improved ethanol production from newspaper, suggesting that this strategy is useful for the selection of the optimal combination of CBMs for each type of biomass [47].

The cellulosome, which is a multi-enzyme complex composed of scaffolding proteins and various cellulosomal enzymes, is produced by some clostridia and plays an important role in efficient degradation of polysaccharides in the plant cell wall. The complex is constructed through the interaction between a cohesin module in the scaffolding protein and a dockerin module in cellulosomal enzymes. Recently, reconstruction of mini-cellulosome on the yeast cell surface has been attempted. The engineered scaffolding protein consisting of three cohesin domains derived from *Clostridium thermocellum*, *Clostridium cellulolyticum*, and *Ruminococcus flavefaciens* was displayed on the *S. cerevisiae* cell surface using the **a**-agglutinin-based display system. The incubation of the scaffolding protein-displaying yeast with three recombinant cellulases (EG, CBH, and BGL) fused with a dockerin domain produced by *Escherichia coli* led to the synergistic hydrolytic degradation of cellulose [48]. Furthermore, a yeast consortium system has been developed, in which four kinds of engineered yeasts are co-cultivated [49]. In this system, construction of the mini-cellulosome was achieved by co-cultivation of a yeast displaying scaffolding protein and yeasts secreting three kinds of dockerin-fused enzymes.

Hemicellulose, including xylan, is also a major component of cellulosic biomass, Furthermore, ethanol production from hemicellulose is important for full utilization of cellulosic biomass. Xylan is hydrolyzed to xylo-oligosaccharides by endo-β-xylanase, and the produced xylo-oligosaccharides are hydrolyzed to d-xylose by β-xylosidase. *S. cerevisiae* co-displaying xylanase II (XYN II) from *T. reesei* and β-xylosidase (XylA) from *A. oryzae* was constructed using the α-agglutinin-based display system, and shown to hydrolyze xylan to xylose [50]. In a further development for ethanol production from xylan, xylose reductase (XR) and xylitol dehydrogenase (XDH) from *Pichia stipitis* and xylulokinase (XK) from *S. cerevisiae* were produced in the XYN II- and XylA-co-displaying yeast. The arming yeast catalyzes simultaneous saccharification and fermentation of xylan [50,51]. In addition to the two-step isomerization of xylose into xylulose, xylose isomerase (XI) is an alternative enzyme that shows great promise, as it is not associated with a cofactor imbalance. Recently, XI from *Clostridium cellulovorans* was successfully displayed and retained activity on *S. cerevisiae*. The constructed XI-displaying yeast could grow in medium containing xylose as the sole carbon source and directly produce ethanol from xylose [52].

Lignin is also a major component of cellulosic biomass, and inhibits cellulose degradation by cellulases, because of its physiological recalcitrancy and its masking the cellulose fibers. Therefore, the removal of lignin from cellulosic biomass is required for efficient degradation. Laccase from white-rot fungus, which participates in several biological pathways including lignin degradation, was displayed on *S. cerevisiae* via the α-agglutinin-based display system. By pretreatment of hydrothermally processed rice straw with laccase-displaying yeast, ethanol production by yeast co-displaying EG II, CBH II, and BGL1 was improved [53].

### 3.2. Bioproduction of Chemicals

Lipase is one of the most applied enzymes in cell surface display on yeast because lipases are used in wide range of industries and the display of lipase is an attractive approach for creating a whole-cell biocatalyst (Figure 2). *R. oryzae* lipase (ROL) and *Candida antarctica* lipase B (CALB) have been extensively studied and are one of the most widely used lipases. ROL was displayed on *S. cerevisiae* by the α-agglutinin-based display system, and the activity of displayed ROL was improved by inserting a spacer peptide with a Gly/Ser repeat between ROL and the cell wall-anchoring domain of α-agglutinin [21,22]. ROL displayed on *S. cerevisiae* showed higher activity than commercially available free and immobilized lipases in organic solvents because the displayed lipase is stabilized by the cell wall [54]. ROL-displaying *S. cerevisiae*, constructed by the Flo1p-based display system, could synthesize methylesters from triglyceride and methanol [28]. In addition, yeast displaying ROL could be used for the optical resolution of (*R,S*)-1-benzyloxy-3-chloro-2-propyl monosuccinate [55]. CALB has also been displayed on *S. cerevisiae* in several studies. Mutated CALB displayed on yeast using the α-agglutinin-based display system has higher thermal stability [56]. Furthermore, CALB-displaying yeasts constructed using the α-agglutinin- or Flo1p-display systems have been used for several ester syntheses with reduced amount of by-products [57,58,59,60]. With regard to lipases, several cell surface display strategies have been attempted in yeasts other than *S. cerevisiae*. In *P. pastoris*, CALB was displayed on the cell surface using the *S. cerevisiae* α-agglutinin-based display system. Compared to CALB-displaying *S. cerevisiae*, the engineered *P. pastoris* showed higher activity, and the ability to synthesize ethyl hexanoate was enhanced [61]. CALB was also displayed on *P. pastoris* via the Sed1p-based display system, and exhibited improved thermal stability [4]. ROL, *Y. lipolytica* lipases, and *Pseudomonas fluorescens* lipase were displayed on *P. pastoris* using the Flo1p-based display system [32,62,63]. Furthermore, *Y. lipolytica* lipase was displayed on *Y. lipolytica* by using the Flo1p-based display system [35].

Isoflavone aglycones, which are bioactive and easily adsorbed by human cells, are hydrolysates of isoflavone glycosides by β-glucosidase. Three kinds of β-glucosidases were independently displayed on *S. cerevisiae* using the α-agglutinin-based display system. Among them, BGL1-displaying yeast could convert isoflavone glycosides into isoflavone aglycones most efficiently [64]. Carnosine and chitosan oligosaccharides also show bioactivities such as antioxidant, antiglycation, cytoplasmic buffering, antitumor, and anticancer properties. The synthesis of carnosine from β-alanine and histidine was achieved through a reverse reaction catalyzed by human carnosinase (CN1)-displaying *S. cerevisiae* in organic solvents and ionic liquids [65]. Cell surface display of chitosanase from *Paenibacillus fukuinensis* enabled the production of chitosan oligosaccharides from chitosan [66]. In *Y. lipolytica*, alginate lyase from *Vibrio* sp. was displayed by fusion with the cell wall-anchoring domain of YlCWP1. The *Y. lipolytica* displaying alginate lyase could hydrolyze poly-β-d-mannuronate, poly-α-l-guluronate, and sodium alginate to produce oligosaccharides [67].

### 3.3. Bioadsorption

Arming technology has been applied to bioadsorption of toxic metal ions and rare-metal ions, leading to bioremediation and resource recovery (Figure 2) [12,13]. The cell surface display of metal-binding proteins/peptides enables the enhanced adsorption and recovery of metal ions on the cell surface. Cell surface adsorption has advantages that are lacking in intracellular accumulation; (i) no requirement for cell disruption for recovery of the adsorbed metal ions; (ii) repeated use of arming yeasts for the further adsorption of metal ions; (iii) rapid and selective adsorption of target metal ions. For the construction of bioadsorbents for bioremediation, toxic metal-binding proteins/peptides such as hexa-His, NP peptides (harboring the CXXEE metal fixation motif of the bacterial Pb^2+^-transporting P1-type ATPases), and metallothionein were displayed on *S. cerevisiae* using the α-agglutinin-based display system [68,69,70,71]. The bioadsorbents constructed by arming technology showed enhanced adsorption of heavy metal ions. Furthermore, cell surface adsorption of metal ions improved cellular tolerance to heavy metal ions [69,71].

Bioadsorption to the cell surface has also been used for adsorption and recovery of rare metal ions. Some of these ions are essential trace metals that play an important role in living cells. For the specific binding and recovery of target metal ions, metal-responsive transcription factors that can bind and dissociate metal ions are displayed and repurposed as cell surface metal-binding proteins. Uptake of molybdenum, one of the rare metals, is regulated by transcription factor ModE that binds molybdate and controls the expression of downstream operon in *E. coli* [72]. Therefore, the full length or *C*-terminal domain of ModE from *E. coli* has been displayed on *S. cerevisiae* via the α-agglutinin-based display system. Molybdate adsorption was achieved using yeast displaying the *C*-terminal domain of ModE, and more than 50% of the molybdate adsorbed on the cell surface could be recovered by papain treatment [73]. Furthermore, a single amino acid mutation (T163Y) of the metal-binding pocket of ModE was efficient to convert it to a selective binder of tungstate, in contrast to the wild-type protein, which binds both tungstate and molybdate. Arming yeast displaying this mutant ModE was shown to selectively uptake tungstate [74].

### 3.4. Bioremediation

To remove or degrade environmental pollutants other than heavy metal ions, arming yeasts for bioremediation have been constructed (Figure 2). For example, to antagonize endocrine disruptors, which are environmental pollutants that perturb natural endocrine function, the ligand-binding domain of the rat estrogen receptor (ERLBD) has been displayed on *S. cerevisiae* using the α-agglutinin-based display system. The ERLBD-displaying yeast was able to bind estrogen-like compounds with an affinity comparable to the native receptor, suggesting the possible application in screening, adsorption, and removal of endocrine disruptor-like chemicals from the environment [75].

Organophosphorus compounds (OPs) are one of the most widely used pesticides, but discharge to the environment is a problem to be solved due to significant threat to public health. Therefore, to degrade OPs using a cell-based system, organophosphorus hydrolase (OPH) from *Flavobacterium* spp. was displayed on *S. cerevisiae* using the α-agglutinin- and Flo1p-based display systems. OPH-displaying yeasts showed hydrolase activity against paraoxon; the activity in the Flo1p-based display system was higher than that in the α-agglutinin-based display system because the active center is located near the *C*-terminal of OPH [76,77]. Furthermore, the development of a biosensor for the sensitive and rapid detection of OPs was achieved by detecting the protons generated by OPs hydrolysis. In this system, additional display of EGFP was performed in OPH-displaying yeast to evaluate the proton generation by the change in fluorescence intensity of EGFP. Together with OPH-dependent hydrolysis, the fluorescence intensity of EGFP was decreased [78]. In another system for OP sensing, the concentration of *p*-nitrophenol produced by OP hydrolysis in OPH-displaying yeast was examined [79].

### 3.5. Other Applications

Other applications of arming technology include biosensors, oral vaccines, antibodies, and stress tolerance. For the non-invasive sensing of environmental changes or monitoring of heterologous protein production, the cell surface display of fluorescent proteins on *S. cerevisiae* was used as a reporter system. The strategy for biosensor construction is that different fluorescent protein variants under the control of different promoters are displayed on the cell surface. GFP display has been placed under the control of the *GAPDH* promoter that is induced in the presence of glucose, whereas BFP was displayed under the control of the *UPR-ICL* promoter from *Candida tropicalis*, which is activated upon the exhaustion of glucose. Therefore, the concentration of intra- or extracellular glucose could be estimated by measuring the fluorescence intensities of GFP and BFP [80]. In addition, monitoring of intra- and extracellular concentrations of phosphate and ammonium ions has been performed by arming yeasts in which the *PHO5* and *MEP2* promoters regulate the genes for cell surface display of ECFP and EYFP, respectively [81]. By using the same *GAL1* promoter for the production of heterologous proteins and cell surface display of EGFP, protein production could be monitored by measuring the fluorescence intensity of EGFP [82].

Construction of an oral vaccine was attempted by displaying antigen on the cell surface of *S. cerevisiae*. 380R antigen from the red sea bream iridovirus (RSIV) was displayed for oral vaccination of cultured marine fish using the α-agglutinin-based display system [83]. In *P. pastoris*, hemagglutinin protein from a highly pathogenic avian influenza (HPAI), subtype H5N1, was displayed with the α-agglutinin-based display system. Oral vaccination of chickens with the hemagglutinin-displaying *P. pastoris* caused the production of virus neutralizing antibodies in the serum [84]. For diagnostics and therapeutics, antibodies and related molecules are attractive targets to be displayed. As a feature of cell surface display system in yeast, produced proteins undergo post-translational modification and efficient disulfide isomerization in a manner that is mechanistically similar to that in mammalian cells. Therefore, hetero-oligomeric proteins such as Fab fragments of catalytic antibodies have been successfully displayed on *S. cerevisiae*, in which the light chain (Lc fragment) of a Fab fragment was displayed on cell surface and the heavy chain (Fd fragment) of a Fab fragment was produced by secretion. As one example, the arming yeast displaying the Fab fragment of hydrolytic antibody 6D9 could catalyze the hydrolysis of a chloramphenicol monoester derivative and showed high stability in binding with a transition-state analog (TSA) [85,86]. In a similar strategy, an engineered Fab fragment, in which mutations were introduced into the Lc fragment to form a catalytic triad, was displayed on *S. cerevisiae*, leading to a higher catalytic activity compared to the wild type Fab fragment [87]. The ZZ domain of protein A from *Staphylococcus aureus* is a repeat of the Z domain that binds to the Fc fragment of human or rabbit IgG. Arming yeast displaying the ZZ domain could be applied to the detection of IgG by enzyme-linked immunosorbent assay (ELISA) and repeated affinity purification of IgG from serum [88].

Arming technology has been also used in molecular breeding of stress-tolerant *S. cerevisiae*. The yeast cell surface was modified by displaying combinatorial random peptides, and acid-tolerant yeast from a yeast library displaying random peptides was successfully selected by culture in acidic conditions. As a result, Scr35 peptide (25 a.a.) was found to enhance the acid tolerance of yeast when displayed on the cell surface [89]. In addition, a combinatorial random protein library was constructed from cDNA of *S. cerevisiae*, and this library was displayed on the yeast cell surface. From the yeast library, nonane-tolerant yeast has been obtained by screening on nonane-overlaid culture medium [90,91]. Cell surface display of leucine-rich peptides has also allowed the creation of yeasts with increased resistance to salt, ethanol, and acetonitrile [92].

## 4. Protein Engineering and Directed Evolution by Arming Technology

### 4.1. Engineering of Enzymes

**Figure 3:**
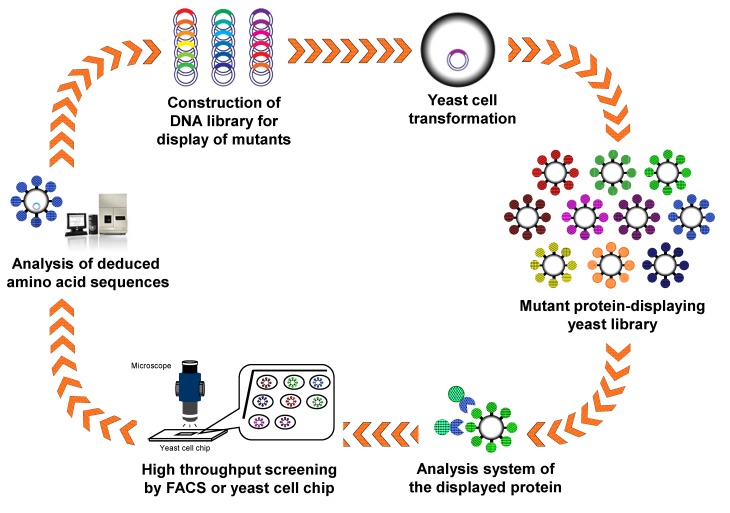
Creation of novel proteins/peptides with improved or developed function by arming technology.

The advantages of arming technology, including direct analysis of target proteins/peptides using intact cells without concentration and purification, are suitable for high-throughput screening of protein/peptide libraries containing random or comprehensive mutations. Therefore, protein engineering has been performed by display of randomly and/or comprehensively mutated proteins/peptides and subsequent high-throughput screening (Figure 3). A combinatorial library of the lid domain of ROL was displayed on the *S. cerevisiae* cell surface. Using a halo assay on soybean oil-containing plates and a assay using fluorescent substrates, lipase mutants with a high substrate specificity toward short-chain substrates and a unique oxyanion hole have been identified from the library [93]. Neurolysin is a metalloendopeptidase that cleaves the bioactive peptide neurotensin. A mutant library of neurolysin constructed by semirational mutagenesis was displayed, and screening was performed using two fluorescence-quenching peptides, the matrix metalloproteinases-2/9- (MMPs-2/9) and MMP-3-specific substrates. As a result, the Y610L mutant of neurolysin was found to show the altered substrate specificity [94]. In addition, the protease inhibitory activity of matrix metalloproteinase-2/9 was improved by arming technology and an automatic single-cell pickup system [6]. Luciferase is also an interesting target for protein engineering, as it plays an important role in the pyrosequencing system of next-generation DNA sequencers. A mutant luciferase library was constructed by arming technology, and interesting mutant luciferases with improved specific activity and dATP discrimination were obtained through only three step-wise screenings [95]. The further technological innovation of the system for single cell analysis and isolation would lead to more efficient protein engineering and directed evolution by arming technology.

### 4.2. Antibody Engineering

Improvement of the affinity of antibodies for target biomolecules is an important and essential challenge in diagnostic and therapeutic development. Single-chain variable fragments (scFv) are those in which the light and heavy chains are linked by a flexible linker. A scFv library was displayed on *S. cerevisiae* by using the **a**-agglutinin-based display system, and the display efficiency was monitored using *N*-terminal or *C*-terminal epitope tags. scFv clones with improved properties could be efficiently isolated from the library through the incubation of scFv-displaying yeast library with fluorescently labeled antigen and the subsequent screening by fluorescently activated cell sorting (FACS) [7]. Furthermore, antibodies to botulinum neurotoxins, carcinoembryonic antigen, CD3 diphtheria toxin, lysozyme, and streptavidin have been improved or developed using the similar strategies based on arming technology [96,97,98,99,100,101]. Thus, arming technology is an attractive methodology for the isolation of antibodies with high affinity and other specific biological functions.

### 4.3. Ligand Screening for Activation of GPCRs

G protein-coupled receptors (GPCRs) are seven transmembrane-domain proteins with the ability to mediate rapid responses to extracellular signals, and they play an important role in many aspects of cellular physiology. Therefore, the design and identification of peptide-ligands for GPCRs are an attractive research area contributing to diagnostics. In the study of ligand-GPCR interactions and identification of ligands against orphan GPCRs, display of peptide ligands specifically on the plasma membrane is important, because ligands displayed only on the cell wall would be unable to access GPCRs on the plasma membrane. Hara *et al.* [36] have developed a display system of peptide-ligand on the yeast plasma membrane for activation of GPCR signaling. First, this system was applied to activation of Ste2 signaling in *S. cerevisiae*, where activation was detected using a Ste2-responsive *FUS1* promoter driving EGFP reporter expression. The α-factor displayed on the plasma membrane was shown to functionally activate the pheromone response pathway. Furthermore, this system has been applied to the activation of human GPCR signaling as well as the yeast pheromone response pathway using chimeric Gα protein. Somatostatin is a naturally occurring gastrointestinal hormone that regulates various endocrine and exocrine processes. After the construction of yeast producing human somatostatin receptor subtype-2 (SSTR2) and chimeric Gα protein, somatostatin was displayed on plasma membrane. The somatostatin displayed on the plasma membrane could activate human SSTR2 in *S. cerevisiae* [37]. This technological platform, namely “PepDisplay” is useful for identification of novel peptide-ligands for heterologously produced GPCRs by membrane display of peptides with random and/or comprehensive sequences and screening based on the activation of reporter genes.

## 5. Conclusions

Arming technology is well established and has been applied to a wide range of research areas, owing to the advantages that are absent in the conventional expression systems. In the construction of whole-cell biocatalysts, cell surface display of enzymes enables bioconversion of the substrate with high-molecular mass that can not enter in the cells, and multistep and synergistic reactions can be achieved on the cell surface by co-displaying multiple kinds of enzymes on the cell surface. Bioadsorption on the cell surface is advantageous in that it does not affect intracellular biological mechanisms, and the adsorbed materials such as metal ions can be easily concentrated and rapidly recovered. Furthermore, arming technology is an innovative molecular tool for protein engineering and directed evolution. The fact that proteins/peptides displayed on the cell surface can be directly, speedily, and conveniently analyzed using intact cells without concentration and purification is suitable for high-throughput screening of a protein/peptide library carrying random and/or comprehensive mutations. Therefore, arming technology is a powerful technology that will facilitate the development of a wide range of biotechnologies and contribute to the production of various kinds of materials for a sustainable society in the future.
